# Gene silencing of the tick protective antigens, *Bm86*, *Bm91* and *subolesin*, in the one-host tick *Boophilus microplus* by RNA interference

**DOI:** 10.1016/j.ijpara.2006.11.005

**Published:** 2007-05

**Authors:** Ard M. Nijhof, Amar Taoufik, José de la Fuente, Katherine M. Kocan, Erik de Vries, Frans Jongejan

**Affiliations:** a Utrecht Centre for Tick-borne Diseases (UCTD), Department of Infectious Diseases and Immunology, Faculty of Veterinary Medicine, Utrecht University, Yalelaan 1, 3584CL Utrecht, The Netherlands; b Department of Veterinary Pathobiology, Center for Veterinary Health Sciences, Oklahoma State University, Stillwater, OK 74078, United States; c Instituto de Investigación en Recursos Cinegéticos IREC (CSIC-UCLM-JCCM), Ronda de Toledo s/n, 13071 Ciudad Real, Spain; d Department of Veterinary Tropical Diseases, Faculty of Veterinary Science, University of Pretoria, Private Bag X04, 0110 Onderstepoort, South Africa

**Keywords:** Bm86, Bm91, Subolesin, RNAi, *Boophilus microplus*, One-host tick

## Abstract

The use of RNA interference (RNAi) to assess gene function has been demonstrated in several three-host tick species but adaptation of RNAi to the one-host tick, *Boophilus microplus*, has not been reported. We evaluated the application of RNAi in *B. microplus* and the effect of gene silencing on three tick-protective antigens: Bm86, Bm91 and subolesin. Gene-specific double-stranded (dsRNA) was injected into two tick stages, freshly molted unfed and engorged females, and specific gene silencing was confirmed by real time PCR. Gene silencing occurred in injected unfed females after they were allowed to feed. Injection of dsRNA into engorged females caused gene silencing in the subsequently oviposited eggs and larvae that hatched from these eggs, but not in adults that developed from these larvae. dsRNA injected into engorged females could be detected by quantitative real-time RT-PCR in eggs 14 days from the beginning of oviposition, demonstrating that unprocessed dsRNA was incorporated in the eggs. Eggs produced by engorged females injected with *subolesin* dsRNA were abnormal, suggesting that subolesin may play a role in embryonic development. The injection of dsRNA into engorged females to obtain gene-specific silencing in eggs and larvae is a novel method which can be used to study gene function in tick embryogenesis.

## 1 Introduction

The cattle tick *Boophilus microplus* is an important pest of cattle in subtropical and tropical regions of the world (Estrada-Pena et al., 2006). Although all *Boophilus* species including *B. microplus* have been reclassified to the genus *Rhipicephalus* (Murrell and Barker, 2003), we maintain use of the previous genus assignment for the purpose of biological clarity. Besides causing direct production losses and leather damage, *B. microplus* transmits several cattle pathogens, including *Babesia bovis*, *Babesia bigemina* and *Anaplasma marginale*. Control of *B. microplus* depends primarily on the use of acaricides or genetically resistant animals. Both approaches have limitations, including development of acaricide resistance, environmental contamination, pesticide residues in food products, the expense of developing new pesticides and the difficulty of producing tick-resistant cattle while maintaining desirable production characteristics (Willadsen, 2004). Other tick control approaches which show promise are the use of biological control agents (reviewed by Samish et al., 2004) and anti-tick vaccines (reviewed by de la Fuente and Kocan, 2003; Willadsen, 2004).

Two commercial vaccines have been developed for control of tick infestations on cattle, TickGARD Plus^®^ in Australia and Gavac^®^ in Cuba. Both are based on the same recombinant antigen named Bm86, a glycoprotein of unknown function which is located predominantly on the surface of midgut digest cells (Gough and Kemp, 1993). This ‘concealed’ antigen is not naturally exposed to the host’s immune system. Lysis of midgut digest cells occurs in ticks that feed on vaccinated cattle, resulting in leakage of blood meal into the tick hemocoel. The overall effect of the vaccine is on engorging female ticks and includes a decrease in the number and weight of replete ticks and oviposition. While Bm86-based vaccines were effective against several other tick species, including *Boophilus annulatus* (Fragoso et al., 1998; Pipano et al., 2003), *Boophilus decoloratus*, *Hyalomma anatolicum anatolicum* and *Hyalomma dromedarii*, they were not effective against *Amblyomma variegatum, Amblyomma cajennense* and *Rhipicephalus appendiculatus* (de Vos et al., 2001; Rodriquez and Jongejan, unpublished data).

Another ‘concealed’ antigen, Bm91, was shown to increase the efficacy of the Bm86 vaccine for *B. microplus* when co-administered (Willadsen et al., 1996). Bm91 is a low-abundance glycoprotein located in the salivary glands and midgut of *B. microplus* (Riding et al., 1994). The protein, a homologue of carboxydipeptidase, shares many biochemical and enzymatic properties with mammalian angiotensin converting enzyme, but its natural substrate has not been identified (Jarmey et al., 1995).

More recently, a protein first labeled 4D8 and now called subolesin was identified through *Ixodes scapularis* cDNA expression library immunisation as a potential tick-protective antigen. Immunisation trials using recombinant *subolesin* caused reductions of larval, nymphal and adult *I. scapularis* infestations (Almazan et al., 2003, 2005a,b). The protein was later found to be conserved among ixodid tick species. Characterisation of its function by RNA interference (RNAi) in *I. scapularis*, *Amblyomma americanum*, *Rhipicephalus sanguineus*, *Dermacentor variabilis* and *Dermacentor marginatus* suggested involvement of this protein in the modulation of blood ingestion and reproduction. Therefore, the generic name “subolesin” was introduced for the 4D8 proteins and “*subA*” for the *subolesin*-encoding gene (de la Fuente et al., 2006a). Gene silencing by RNAi of *subA* and *Rs86*, the homologue of *Bm86*, in *R. sanguineus*, revealed a synergistic effect in which the expression of both genes was silenced and resulted in decreased tick attachment, feeding and oviposition (de la Fuente et al., 2006c).

RNAi is a conserved post-transcriptional gene-silencing mechanism present in ticks and a wide range of eukaryotes in which double-stranded RNA (dsRNA) triggers a sequence-specific degradation of cognate mRNAs. It has been an effective tool to study the function of tick proteins at the tick–pathogen interface in a number of three-host tick species such as in *I. scapularis* which transmits *Anaplasma phagocytophilum* and *Borrelia burgdorferi* (Pal et al., 2004; Ramamoorthi et al., 2005; Sukumaran et al., 2006). RNAi was used to study the function of several tick salivary gland proteins involved in feeding of *A. americanum* (Aljamali et al., 2003; Karim et al., 2004), *Haemaphysalis longicornis* (Miyoshi et al., 2004) and *I. scapularis* (Narasimhan et al., 2004). The inducer of RNAi, dsRNA, is injected into nymphal or adult ticks which are then allowed to feed normally. Capillary feeding of dsRNA (Soares et al., 2005) or incubation of isolated tick tissues with dsRNA (Aljamali et al., 2003; Karim et al., 2005) are other methods used successfully to silence genes in ticks. These studies suggest that RNAi is systemic and effects gene silencing throughout the tick.

In one-host *Boophilus* ticks, with all life stages feeding and molting on the same host, alternative strategies are required to conduct gene silencing by RNAi as compared with three-host tick species which spend their non-parasitic life stages off-host. Herein, we examined two methods of dsRNA delivery and its effect on the one-host tick *B. microplus*: (i) injection of dsRNA into freshly molted females and (ii) injection of dsRNA into engorged females. The latter method caused gene-specific silencing in the oviposited eggs and larvae that hatched from these eggs. We believe this is the first report of the silencing of the expression of Bm86, Bm91 and subolesin in *Boophilus* ticks as quantified by real-time RT-PCR using two routes of dsRNA delivery.

## 2 Materials and methods

### 2.1 Experimental animals

Three Holstein–Friesian calves, 5 months of age (#7793, #7794 and #7799), were used. All animals had no previous exposure to ticks. All tick feedings were approved by the Animal Experiments Committee (DEC) of the Faculty of Veterinary Medicine, Utrecht University (DEC No. 0111.0807).

### 2.2 Ticks and tick feeding

*Boophilus microplus* ticks originating from Mozambique were provided by ClinVet International (Pty), Bloemfontein, South Africa. The ticks were subsequently maintained on cattle at our tick rearing facility. Larvae were kept off-host at 20 °C with 95% relative humidity. Patches used for tick feeding with inner dimensions of 60 × 85 mm and sewn to an open cotton bag were glued to the shaved back of calf #7793 and #7794 using Pattex^®^ contact glue (Henkel Nederland, Nieuwegein, The Netherlands). A batch of larvae eclosed from 1500 mg of pooled eggs oviposited by 25 females (approximately 24,000 larvae), was divided on day 0 between two patches on calf #7794. Since males appear earlier from the nymphal stage than females, approximately 500 unfed males were collected on days 13 and 14 and 600 unfed females on days 14 and 15 and incubated at 27 °C with 95% relative humidity. Freshly molted females were subjected to injection of dsRNA on day 15 as described below. For gene silencing in engorged females and their progeny, 25 engorged females with an average weight of 261 mg (248–272 mg) fed on calf #7794 were collected on day 21. Larvae which hatched from eggs laid by mock-injected, *Bm86*- and *Bm91*-dsRNA injected engorged females were fed in three patches on calf #7799.

### 2.3 RNA extraction and synthesis of tick cDNA for dsRNA preparations

The viscera of five partially fed *B. microplus* females were dissected in ice-cold PBS and immediately stored in 1 ml Tri reagent (Sigma–Aldrich, Zwijndrecht, The Netherlands) at −80 °C. Total RNA was isolated and subsequently purified using the Nucleospin RNA II kit (Macherey-Nagel, Düren, Germany) in accordance with the reagent and kit manufacturer’s directions. Total RNA concentration was determined spectrophotometrically and the material was stored at −80 °C before use. Complementary DNA was made with the Revertaid first strand cDNA synthesis kit (Fermentas, St. Leon-Rot, Germany) in accordance with the manufacturer’s protocol using random hexamer primers. Control reactions were performed using the same procedures but without RT as a control for DNA contamination in the RNA preparations.

### 2.4 Cloning and sequencing of the B. microplus subolesin gene

Cloning and sequencing of the *subA* gene from the Mozambiquan *B. microplus* strain was performed as described elsewhere (de la Fuente et al., 2006a). The sequence has been submitted to GenBank and can be retrieved under Accession No. DQ923495.

### 2.5 dsRNA synthesis

Oligonucleotide primers containing T7 promotor sequences at the 5′-end for in vitro transcription and synthesis of dsRNA were used to PCR-amplify cDNA encoding *B. microplus Bm86* (421 bp), *Bm91* (417 bp) and *subolesin* (381 bp). All oligonucleotide primers used in this study were synthesised by Isogen Life Science, IJsselstein, The Netherlands and their sequences are shown in Table 1. PCR products were purified using the GfX PCR purification kit (Amersham) and used as templates to produce dsRNA using the T7 Ribomax Express RNAi system (Promega, Leiden, The Netherlands). dsRNA aliquots were stored at −80 °C until used.

### 2.6 Injection of ticks with dsRNA

Freshly molted females were placed on double-sided sticky tape with the ventral sides upwards and injected into the anal aperture with 0.5 μl *Bm86*, *Bm91* or *subolesin* dsRNA alone or a combination of *Bm86* and *subolesin* dsRNA (6–9 × 10^11^ molecules/μl) using a 10 μl syringe with a 33 G needle (Hamilton, Bonaduz, Switzerland) mounted on a MM3301-M3 micromanipulator (World Precision Instruments (WPI), Berlin, Germany) and connected to an UMPII syringe pump (WPI). The tip of a 27 G needle was used to slightly pierce the anal aperture before the 33 G needle was inserted. The dsRNA was dissolved in injection buffer (10 mM Tris–HCl, pH 7 and 1 mM EDTA). A control group was injected with injection buffer alone. The ticks were placed in an incubator at 27 °C with 95% relative humidity for 3–10 h following injection, before they were examined for mortality and placed in five separate patches, one for each group, on calf #7793. One hundred male ticks were placed in each patch simultaneously with the injected females. The ticks were checked twice daily and collected when they dropped from the host. Ticks still attached 14 days after the dsRNA-injection (day 29 after application of the larvae) were removed manually. All ticks were weighed separately within 1 h after collection and stored individually in 1.5 ml Eppendorf tubes with pierced lids at 27 °C and 95% relative humidity for oviposition. For the second experiment, engorged *B. microplus* females were injected with 5 μl of *Bm86*, *Bm91* or *subolesin* dsRNA (1–2 × 10^12^ molecules/μl) or injection buffer alone in the right spiracular plate within 6 h after dropping off the host, using the same methods as described above, or left uninjected. They were stored individually in 2 ml Eppendorf tubes with pierced lids in an incubator at 27 °C and 95% relative humidity. Eggs were removed daily and each daily egg batch was stored separately under the same conditions.

### 2.7 Analysis to confirm gene silencing by quantitative RT-PCR

Viscera was dissected from five females of each dsRNA-injected or mock-injected group after 6 days of feeding. Total RNA was isolated from these samples using Tri reagent and subsequently purified using the Nucleospin RNA II kit in accordance with the reagent and kit manufacturer’s directions. Total RNA was isolated from 100 mg eggs (14 days after injection), 50 mg larvae (at 6 days and 5 weeks after hatching) laid by/eclosed from the dsRNA- and mock-injected engorged females, 50 mg larvae at 10 weeks after hatching laid by the dsRNA- and mock-injected unfed females and from the dissected viscera of five females and five males which developed from 7-week-old larvae fed on animal #7799 using the same methods. cDNA from 1 μg of RNA (adults, eggs and 6-day-old larvae) and 0.3 μg of RNA (5-week-old larvae) was prepared using the Revertaid first strand cDNA synthesis kit (Fermentas) using random hexamer primers in accordance with the manufacturer’s protocol. All samples were analyzed for transcription of target genes by quantitative real-time RT-PCR using primers Bm86h-F6 and Bm86h-R4, amplifying a 117 bp section of the *Bm86* gene; Bm91-F2 and Bm91-R3, amplifying a 129 bp section of the *Bm91* gene and Bm-subA-F2 and Bm-subA-R2, amplifying a 166 bp section of the *subolesin* gene. Tick β-actin was included as a control and used for normalisation. A 126 bp fragment was amplified using primers Actin-F2 and Actin-R. All primer combinations amplified a different part of the targeted genes than the sections which were used for dsRNA synthesis, circumventing re-amplification of any unprocessed dsRNA. Twenty-five microlitres of real-time PCRs were performed using the Quantitect SYBR green PCR kit in accordance with the manufacturer’s protocol (Qiagen, Venlo, The Netherlands) on an iCycler real-time detection system (Bio-Rad Laboratories, Veenendaal, The Netherlands). Real-time PCR data were analyzed by iCycler IQ software version 1.0.

### 2.8 Analysis to check for the presence of dsRNA in eggs

cDNA from 1 μg of total RNA of eggs 14 days post-oviposition was screened by quantitative real-time RT-PCR for the presence of non-processed *Bm86*, *Bm91* and *subolesin* dsRNA to see whether the injected dsRNA was incorporated into the eggs and could be re-amplified. Oligonucleotide primers located within the region used for dsRNA synthesis of the *Bm86, Bm91* and *subolesin* genes were used for this purpose. The following primer combinations were used: Bm86-F7 and Bm86h-R3, amplifying a 121 bp section of the *Bm86* gene, primers Bm91-F3 and Bm91-R1, amplifying a 128 bp region of the *Bm91* gene, primers BmsubA-F2 and BmsubA-R1, amplifying a 121 bp section of the *subolesin* gene. Real-time RT-PCR conditions were identical to those used to confirm gene silencing.

### 2.9 Statistical analysis

Statistical analysis of data from two quantitative RT-PCR experiments, the weights of ticks after feeding and oviposited egg masses, was performed using Microsoft Excel and consisted of an unpaired *t*-test with unequal variances. Tick mortality was compared between the dsRNA- and mock-injected ticks by *χ*^2^-test. *P* values of 0.05 or less were considered statistically significant.

## 3 Results

### 3.1 Cloning and sequencing of the subolesin gene from B. microplus

The *subolesin* gene (*subA)* from the Mozambiquan *B. microplus* strain was cloned and sequenced. This gene was found to be 99–100% identical to *subA* from *B. microplus* strains from Mexico and Brazil (de la Fuente et al., 2006a; our unpublished data).

### 3.2 RNAi in freshly molted B. microplus females

**Fig. 1:**
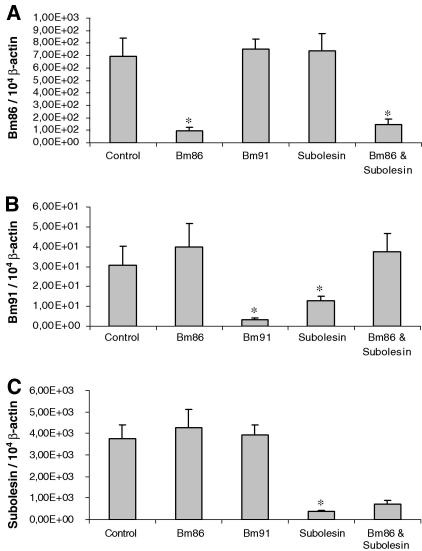
Quantitative real-time RT-PCR analysis showing the relative *Bm86* (A), *Bm91* (B) and *subolesin* (C) transcript levels in the viscera of five partially fed females, 6 days after they were injected with injection buffer alone (Control), *Bm86*-, *Bm91*-, or *subolesin*-double-stranded RNA (dsRNA) or a combination of *Bm86*- and *subolesin*-dsRNA, and fed on calf #7793. The data represent mean values + SD, normalised relative to β-actin transcript levels. Asterisk (*) denotes the difference compared with the control injected group is significant as determined by Student’s *t*-test (*P* < 0.01).

Five groups consisting of 120 freshly molted *B. microplus* females were each injected with 0.5 μl of injection buffer in the following groups: (i) injection buffer alone, (ii) *Bm86*, (iii) *Bm91*, (iv) *subolesin* and (v) both *Bm86* and *subolesin*-dsRNA. An average of 81 (76–82; 32.8% overall mortality) females were alive in each group 3–10 h following injection. These ticks were subsequently fed together on a calf with an excess of *B. microplus* males until the females became replete or for a maximum of 14 days. Tick weight after engorgement or manual removal, mortality rate, egg mass and hatching rate is presented in Table 2. A significant decrease in tick weight and oviposited egg mass, together with a higher mortality rate, was observed in the *subolesin* dsRNA injected groups compared with the control group (*P* < 0.01). Hatching rates were uniformly constant in the control, *Bm86-* and *Bm91-*dsRNA-injected groups (>90%), while in the ticks injected with *subolesin* dsRNA the hatching rate was lower (<20%). Gene silencing was confirmed by quantitative real-time RT-PCR (Fig. 1). The normalised transcript level of *Bm86* was reduced with 86% in the *Bm86*-dsRNA-injected ticks and with 79% in the combined *Bm86*/*subolesin*-dsRNA-injected ticks, compared with the normalised transcript level of the mock-injected group (Fig. 1A). For *Bm91*, the normalised transcript level was reduced with 90% in the *Bm91* RNAi-silenced ticks compared with the control ticks. A significant decrease in the *Bm91* transcript level of 58% was observed in the *subolesin* dsRNA-injected ticks (*P* < 0.01) as well, but this reduction was not observed in the combined *Bm86*/*subolesin*-dsRNA-injected ticks (Fig. 1B). Normalised transcript levels of *subolesin* in the *subolesin* dsRNA- and combined *Bm86*/*subolesin* dsRNA-injected ticks compared with the control group were reduced with 90% and 80%, respectively (Fig. 1C). No differences were observed in the normalised *Bm86* and *Bm91* transcript levels of 10-week-old larvae which hatched from the *Bm86*- and *Bm91*-dsRNA-injected ticks compared with the mock-injected ticks (results not shown).

### 3.3 RNAi in engorged B. microplus females

**Fig. 2:**
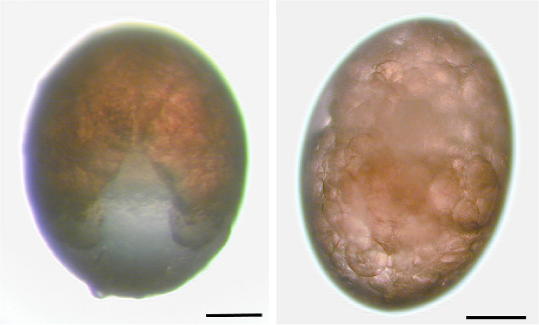
Representative *Boophilus microplus* eggs from females injected with injection buffer alone (left) showing normal development, or *subolesin* double-stranded RNA showing an undifferentiated mass (right). Photographs were taken 20 days after injection of the engorged females and 17 days after oviposition. Bar = 0.1 mm.

Five groups of five engorged females each with an average weight of 261 mg (248–272 mg) were injected with 5 μl of *Bm86* dsRNA, *Bm91* dsRNA, *subolesin* dsRNA, injection buffer alone or left uninjected within 6 h after dropping from the host. No reflux of the injected solution or hemolymph was observed from the puncture when the needle was gently withdrawn. Oviposition began in all groups within 3 days, except for one tick from the control-injected group and another tick from the *Bm91* dsRNA-injected group which did not lay any eggs. The course of oviposition was not significantly influenced by the injection of dsRNA (Table 3), and dried or shriveled eggs were not observed, indicating that all eggs were successfully coated with a secretion from Gené’s organ. Interestingly, nearly all (>99.4%) eggs oviposited by engorged females injected with *subolesin* dsRNA showed an aberrant phenotype compared with those from the other groups. A typical example is shown in Fig. 2. Development of embryos in these eggs was not observed while many undifferentiated cells with some yolk cells were seen in Giemsa-stained egg crush smears. Most eggs did not hatch and eventually dried up and shriveled after 6–7 weeks of incubation at 27 °C/95% relative humidity. The few eggs from this group which did develop and hatched normally (<0.6%) were all laid during the first day of oviposition.

**Fig. 3:**
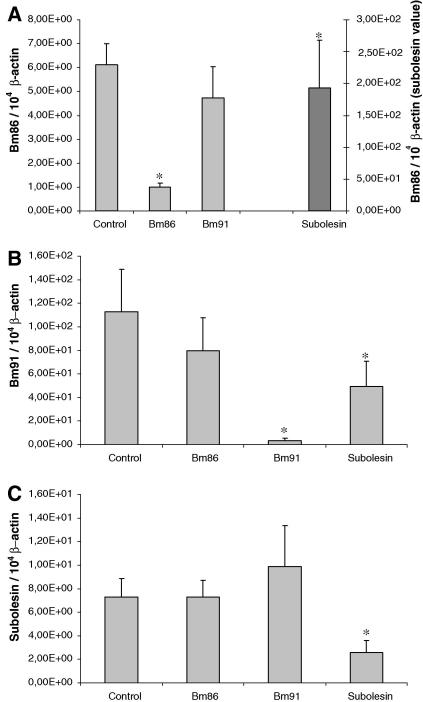
Quantitative real-time RT-PCR analysis showing the relative *Bm86* (A), *Bm91* (B) and *subolesin* (C) transcript levels in eggs originating from mock-injected, *Bm86*-, *Bm91*- or *subolesin*-double stranded RNA-injected engorged females. The data represent mean values + SD, normalised relative to β-actin transcript levels. Asterisk (*) denotes the difference compared with the control injected group is significant as determined by Student’s *t*-test (*P* < 0.01).

A 64% decrease of *subolesin* transcript levels was found in eggs from the *subolesin* dsRNA-injected engorged females (Fig. 3C). Interestingly, a 30-fold increase in the *Bm86* transcript level and a 56% decrease in *Bm91* transcript level were also found in these aberrant eggs (Figs. 3A and B). An 84% decrease in the number of *Bm86* copies was observed in eggs laid by the engorged females injected with *Bm86* dsRNA (Fig. 3A) and the transcript level of *Bm91* was reduced by 97% in eggs from the *Bm91* dsRNA-injected engorged females compared with the mock-injected control group (Fig. 3B), confirming gene-specific silencing in the eggs of dsRNA-injected engorged females.

**Fig. 4:**
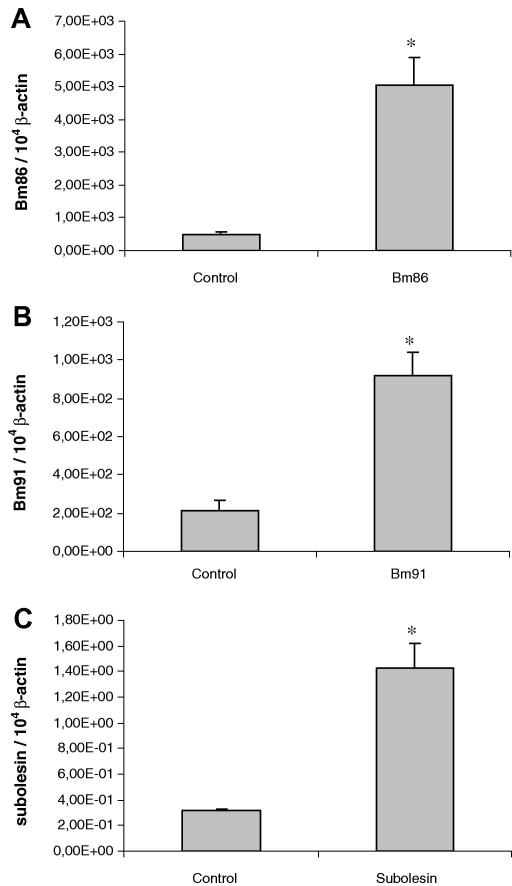
Quantitative real-time RT-PCR analysis using primers located within the double-stranded RNA (dsRNA) region showing the relative *Bm86* (A), *Bm91* (B) and *subolesin* (C) transcript levels in eggs originating from *Bm86*-(A), *Bm91*-(B), or *subolesin*-(C) dsRNA-injected engorged females compared with levels in the mock-injected control group. The data represent mean values + SD, normalised relative to β-actin transcript levels. Asterisk (*) denotes the difference compared with the control injected group is significant as determined by Student’s *t*-test (*P* < 0.01).

Injected dsRNA could be re-amplified from the eggs of dsRNA-injected engorged females when primers located within the dsRNA sections were used, instead of primers located downstream of the dsRNA region which were used to demonstrate gene silencing (Table 1). Results of quantitative real-time RT-PCR performed with these primers showed levels of *Bm86, Bm91* and *subolesin* which were 10.5, 4.3 and 4.5 times higher, respectively, in the *Bm86, Bm91* and *subolesin* injected groups than levels found in the mock-injected group (Fig. 4).

**Fig. 5:**
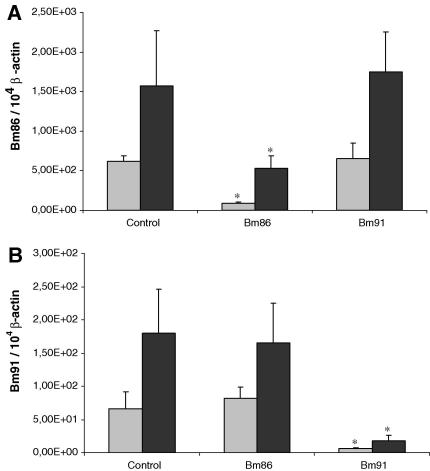
Quantitative real-time RT-PCR analysis showing the relative *Bm86* (A) and *Bm91* (B) transcript levels in 6-day-old larvae (grey bars) and 5-week-old larvae (black bars) originating from mock-injected, *Bm86*- or *Bm91*-double-stranded RNA-injected engorged females. The data represent mean values + SD, normalised relative to β-actin transcript levels. Asterisk (*) denotes the difference compared with the control injected group is significant as determined by Student’s *t*-test (*P* < 0.01).

Gene silencing was observed in larvae 6 days after hatching; a decrease of 86% in *Bm86* transcript level and of 91% in *Bm91* transcript level in the *Bm86*- and *Bm91* dsRNA-injected groups, respectively (Figs. 5A and B, grey bars). *Subolesin* transcript levels were not measured in the few larvae which hatched from the *subolesin* dsRNA-injected females due to their small number. Quantitative real-time RT-PCR analysis of RNA extracted from larvae 5 weeks after hatching (9 weeks after the initial injection of engorged females with dsRNA) revealed that genes remained silenced in both *Bm86*- and *Bm91*-silenced groups (Figs. 5A and B, black bars). This effect diminished over time, in particular for the *Bm86-*silenced group, in which transcript levels were now 67% lower compared with the control group. *Bm91* transcript levels were still 90% lower in the *Bm91*-silenced group compared with the control group. When 7-week-old larvae from these three groups were fed in separate patches on a calf and total RNA from the viscera of adults which had developed from these larvae was analyzed by quantitative real-time RT-PCR, gene silencing was not observed (results not shown).

## 4 Discussion

The production characteristics of the *subolesin*-silenced female *B. microplus* ticks corresponded with previous results from *subolesin* RNAi studies in other ixodid tick species, and typically resulted in decreased tick and egg mass weights and high mortality (de la Fuente et al., 2005, 2006a,c). The synergistic effect of combined silencing of the *Bm86* and *subolesin* gene reported previously in *R. sanguineus* (de la Fuente et al., 2006c) could not be confirmed for *B. microplus* (Table 2).

Significant changes in the production characteristics of the *Bm86*- and *Bm91*-silenced females were not observed, suggesting that the deleterious effect on ticks feeding on Bm86- or Bm91-vaccinated cattle is not caused by a loss of function of the Bm86 or Bm91 protein alone. In fact, the protective effect of Bm86-based vaccines is through gut damage mediated by anti-Bm86 antibodies during tick feeding (Willadsen et al., 1989). These results suggest that the protection mechanisms of Bm86 and subolesin-based vaccines are different and may contribute to the increased efficacy of Bm86 and subolesin combined vaccines.

Quantitative real-time RT-PCR performed on samples taken 6 days after dsRNA injection and feeding resulted in a decrease of 79–90% of the targeted gene transcript level compared with the mock-injected group. This result was comparable with previous semi-quantitative measurements of the gene silencing effect by dsRNA-injection in *A. americanum.* In this RNAi study, a decrease of ∼90% to 50% in *cystatin* transcript level was observed from 24 h to 9 days of feeding (Karim et al., 2005). In the present study, although gene silencing was specific in the *Bm86* and *Bm91* dsRNA-injected groups, *subolesin* dsRNA injection resulted in both significantly decreased *subolesin* as well as *Bm91* expression levels. This effect was not observed in the combined *Bm86*/*subolesin* silenced group, which may be explained by a slight difference in midgut:salivary gland ratio in the dissected viscera between the groups. Since *Bm91* is present in relatively high concentrations in salivary glands compared with midgut (Riding et al., 1994), a shift in the midgut:salivary gland ratio of the dissected viscera in favor of midgut tissue could result in the lower *Bm91* expression levels we found in this specific sample. Alternatively, the effect of silencing subolesin expression may affect the expression of other genes. The pleiotropic effect on tick tissues in which subolesin expression has been silenced suggests that this gene may be involved in the regulation of multiple pathways in ticks (de la Fuente et al., 2006a).

Injection of pilocarpine solution into the hemocoel via the spiracular plate of engorged ticks has been described for inducement of salivation in engorged *B. microplus* ticks (Bechara et al., 1988). These openings of the tracheae are sclerotised structures located posterior to the fourth pair of legs. Their rigid structure allows for puncturing and injection of small quantities of fluid by a fine needle without subsequent reflux of the injected solution, hemolymph or tissue. When we injected *Bm86*, *Bm91* or *subolesin* dsRNA into the hemocoel of engorged females, the course of oviposition appeared to be unaffected. Only one *Bm91* dsRNA-injected female and one mock-injected female tick died before ovipositing. Interestingly, embryogenesis was undisturbed in eggs oviposited by the *Bm86* dsRNA-, *Bm91* dsRNA- and mock-injected engorged females, but an aberrant development was seen in the majority of egg masses oviposited by the *subolesin* dsRNA-injected engorged females. This egg phenotype has not been described previously and suggests that *subolesin* plays a role in embryonic development. When total RNA isolated from these aberrant eggs was analyzed by real-time RT-PCR, significantly higher *Bm86* levels and decreased *Bm91* and *subolesin* levels were found. Again, these results suggest that subolesin may be a regulator of transcription in ticks.

Injected dsRNA was detected in eggs from dsRNA-injected engorged females by real-time RT-PCR using primers located within the dsRNA section, indicating that unprocessed dsRNA is incorporated in the eggs. The suggested route of incorporation of exogenously produced yolk directly from the hemolymph into oocytes (Saito et al., 2005) may be followed for the incorporation of dsRNA into oocytes as well. Further experiments are needed to determine whether this dsRNA forms a reservoir of mobile silencing signals inducing gene-specific silencing in eggs, or whether small interfering RNAs are responsible for this effect. Other possible routes for the mobile silencing signal to enter the oocyte are through the pedicel in the ovary or, once the oocyte has ovulated, by contact with cells from the genital tract.

Some eggs (<0.6%) found in the batches laid during the first day of oviposition by *subolesin* dsRNA-injected engorged females, hatched normally. It is likely that these eggs developed to a stage which was not accessible by the dsRNA prior to injection of the dsRNA. They may have been ovulated eggs which were not in direct contact with the hemolymph or ones in which the shell was hardened during the oocyte passage through the oviduct and thus became impermeable to dsRNA during this process (Diehl et al., 1982).

After dsRNA was injected into the body cavity of *B. microplus*, the gene silencing effect spread throughout the organism and its progeny. This systemic RNAi has been associated with the sid-1 protein, a transmembrane protein which enables passive cellular uptake of dsRNA (Winston et al., 2002; Feinberg and Hunter, 2003). Unfortunately, our attempts to detect a *B. microplus sid-1* homologue, as described previously in studies on presence of a *sid-1* homologue in grasshopper species *Schistocerca americana*, were not successful (data not shown) (Dong and Friedrich, 2005). The only protein currently present in the *B. microplus* expressed sequence tag (EST) database (Guerrero et al., 2005) which is associated with the RNAi machinery is the nuclease Argonaute-2 (Ago-2), the central catalytic component of the RNA-induced silencing complex (RISC) in mammals and *Drosophila* (Liu et al., 2004; Miyoshi et al., 2005). Homologues of other RNAi-associated proteins such as Dicer, which is responsible for the cleavage of exogenous long dsRNA into short interfering RNA (siRNA), remain to be identified in *B. microplus* and other tick species as well.

RNAi described herein provides an important tool to screen for tick-protective antigens in this one-host tick species, *B. microplus* (de la Fuente et al., 2005) and allowed for characterisation of the effect and function of tick protective antigens, as well as the role of genes involved in tick–host–pathogen interactions and the transmission of tick-borne pathogens (de la Fuente et al., 2006b). Initiation and completion of the *B. microplus* genome sequencing project would greatly enhance these kinds of studies (Guerrero et al., 2006), as well as the availability of the genomes from cattle and the pathogens transmitted by *B. microplus*, most notably: *A. marginale* (Brayton et al., 2005), *B. bigemina* and *B. bovis*, which are currently being sequenced. Although sequences of the dsRNAs used in this study do not contain any significant overlap with other known *B. microplus* genes, the possibility of off-target gene silencing effects cannot be excluded due to the limited amount of sequence data available. Availability of the complete *B. microplus* genome sequence data will facilitate screening for potential off-target effects. These can subsequently be minimised by avoiding the use of dsRNAs or siRNAs containing sequences which are present in multiple genes.

The effect of silencing tick genes suggested to be involved in embryogenesis such as *vitellin degrading cysteine endopeptidase* (Seixas et al., 2003), in the transovarial transmission of *Babesia* spp. or genes associated with acaricide resistance, which is measured in larvae by the Larval Packet Test (Li et al., 2003), could be studied using our method to silence genes in *B. microplus* embryos and larvae by injecting engorged females with dsRNA. These experimental approaches are more efficient and less labour intensive than the three other dsRNA delivery approaches into oocytes and embryos using microinjection (Wargelius et al., 1999), transgenic RNAi (Tavernarakis et al., 2000) and electroporation (Grabarek et al., 2002).

## Figures and Tables

**Table 1:** List of oligonucleotide primers used and the purpose for which they were used in this study

Primer	Sequence (5′ → 3′)	Purpose
Bm86h-F3T7	GTAATACGACTCACTATAGGTGCTCTGACTTCGGGAA	*Bm86* dsRNA synthesis
Bm86h-R3T7	GTAATACGACTCACTATAGGTCGCAGAG(AG)TC(TC)TTGCA	*Bm86* dsRNA synthesis
Bm91h-F1T7	GTAATACGACTCACTATAGGCCAACATCAC(GC)GA(GT)TACAAC	*Bm91* dsRNA synthesis
Bm91h-R1T7	GTAATACGACTCACTATAGGG(AT)GACGCTGCTTC(GA)TTGG	*Bm91* dsRNA synthesis
BmsubA-FT7	TAATACGACTCACTATAGGGTACTACATGACTGGGACCCCTTGCAC	*subolesin* dsRNA synthesis
BmsubA-RT7	TAATACGACTCACTATAGGGTACTCTGTTCTGCGAGTTTGGTAGATAG	*subolesin* dsRNA synthesis
Bm86h-F6	CTGC(GA)ACAGAAAT(CT)GAAGAAGA	Measure *Bm86* transcript level
Bm86h-R4	GC(GA)CACT(GT)GAACCA(GA)AAGA	Measure *Bm86* transcript level
Bm91-F2	T(GA)TTGGACAAGTGGCG(GC)T	Measure *Bm91* transcript level
Bm91-R3	AAGAAGGACTCGTTGCGCT	Measure *Bm91* transcript level
Bm-subA-F2	GAGACCAGCCCCTGTTCA	Measure *subolesin* transcript level
Bm-subA-R2	CCGCTTCTGAATTTGGTCG	Measure *subolesin* transcript level
Actin-F2	GACATCAAGGAGAAGCT(TC)TGC	Measure *β*-actin transcript level
Actin-R	CGTTGCCGATGGTGAT(GC)	Measure *β*-actin transcript level
Bm86-F7	ACGGATGGGTTTATTGGC	Measure *Bm86* transcript level, located within dsRNA region
Bm86h-R3	TCGCAGAG(AG)TC(TC)TTGCA	Measure *Bm86* transcript level, located within dsRNA region
Bm91-F3	GGAATATGAAGGAAGTTGGC	Measure *Bm91* transcript level, located within dsRNA region
Bm91-R1	G(AT)GACGCTGCTTC(GA)TTGG	Measure *Bm91* transcript level, located within dsRNA region
BmsubA-F2	GAGACCAGCCCCTGTTCA	Measure *subolesin* transcript level, located within dsRNA region
BmsubA-R1	CTGTTCTGCGAGTTTGGTAGATAG	Measure *subolesin* transcript level, located within dsRNA region

**Table 2:** Tick weight, mortality after feeding, egg mass weight and egg hatching rate in double-stranded RNA (dsRNA)-injected *Boophilus microplus* ticks, injected as freshly molted females

Group (*n*)	Number of ticks	Tick weight^a^ (mg)	Mortality^b^ (%)	Eggs/tick^c^ (mg)	Hatching rate^d^ (%)
Control	82	297 ± 71	27	139 ± 63	>90
*Bm86*	82	276 ± 62	23	108 ± 60	>90
*Bm91*	76	277 ± 67	16	125 ± 67	>90
*Subolesin*	81	75 ± 80^*^	46^**^	1 ± 7^*^	<20^*^
*Bm86* and *Subolesin*	82	42 ± 42^*^	59^**^	0^*^	ND

ND, not determined.

a Ticks which completed feeding and those removed after day 29 (14 days after mock- or dsRNA injection) were weighed individually and average ± SD calculated and compared between dsRNA and mock-injected control ticks, using the Student’s *t*-test with unequal variance (*P < 0.01).

b Tick mortality was evaluated as the ratio of dead female ticks after day 29 (14 days after mock- or dsRNA injection) to the total number of female ticks placed on the animal, and was compared between dsRNA and mock-injected control ticks by *χ*^2^-test (**α < 0.025).

c The egg mass oviposited by each tick was weighed individually and average ± SD calculated and compared between dsRNA and mock-injected control ticks using Student’s *t*-test with unequal variance (*P < 0.01).

d The hatching rate was determined 6 weeks post oviposition and compared between control injected and dsRNA-injected ticks using the Student’s *t*-test with unequal variance (*P < 0.01).

**Table 3:** Tick weight, egg mass weight and egg hatching rate, of engorged *Boophilus microplus* females injected with double-stranded RNA (dsRNA)

Group	Tick weight^a^ (mg)	Eggs/tick^b^ (mg)	Hatching rate^c^ (%)
Uninjected	258 (248–266)	159 (149-167)	>90
Control injected	260 (251–272)	159 (152–168)	>90
*Bm86*	263 (252–270)	159 (149–167)	>90
*Bm91*	263 (252–273)	143 (133–158)	>90
*Subolesin*	262 (253–271)	151 (142–160)	0.6^*^

a Replete *B. microplus* ticks were collected and weighed individually before injection with dsRNA, all within 6 h after dropping of the host. The average weight and variation (between parentheses) of each group are shown.

b The egg mass oviposited by each tick was weighed individually. The average egg mass and variation (between parentheses) was calculated and compared between uninjected and injected ticks using the Student’s *t*-test with unequal variance. No significant statistical differences were observed (*P* > 0.05).

c The hatching rate was determined 6 weeks post oviposition and compared between uninjected and injected ticks by using the Student’s *t*-test with unequal variance (*P < 0.01).
